# Femtosecond electron imaging of defect-modulated phonon dynamics

**DOI:** 10.1038/ncomms11230

**Published:** 2016-04-15

**Authors:** Daniel R. Cremons, Dayne A. Plemmons, David J. Flannigan

**Affiliations:** 1 Department of Chemical Engineering and Materials Science, University of Minnesota, 421 Washington Avenue SE, Minneapolis, Minnesota 55455, USA

## Abstract

Precise manipulation and control of coherent lattice oscillations via nanostructuring and phonon-wave interference has the potential to significantly impact a broad array of technologies and research areas. Resolving the dynamics of individual phonons in defect-laden materials presents an enormous challenge, however, owing to the interdependent nanoscale and ultrafast spatiotemporal scales. Here we report direct, real-space imaging of the emergence and evolution of acoustic phonons at individual defects in crystalline WSe_2_ and Ge. Via bright-field imaging with an ultrafast electron microscope, we are able to image the sub-picosecond nucleation and the launch of wavefronts at step edges and resolve dispersion behaviours during propagation and scattering. We discover that the appearance of speed-of-sound (for example, 6 nm ps^−1^) wavefronts are influenced by spatially varying nanoscale strain fields, taking on the appearance of static bend contours during propagation. These observations provide unprecedented insight into the roles played by individual atomic and nanoscale features on acoustic-phonon dynamics.

## Abstract (web summary)

Phonons—crystal lattice vibrations—interact with crystal defects on the nanometre spatial scale and femtosecond timescale. Here, the authors demonstrate direct, real-space imaging of the nucleation, emergence and dispersion of single-phonon wavefronts at individual atomic-scale defects in semiconductors.

## Graphical abstract

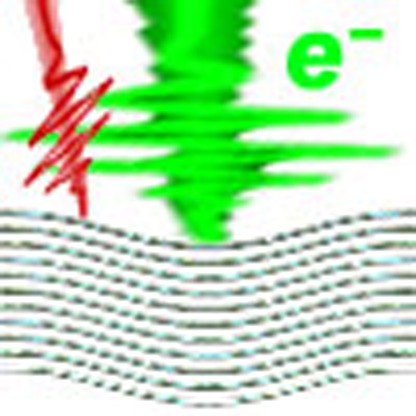

Phonons—quantized elastic oscillations—are at the core of innumerable atomic-scale physical processes and mesoscale phenomena, from structural variations associated with lattice fluctuations, phase transitions and bond modulation^1^,^2^,^3^,^4^,^5^ to electromagnetic and electronic properties, including electric susceptibility and electron mobility^6^,^7^,^8^,^9^,^10^. Consequently, the atomic-scale manipulation and control of phonon modes has been proposed and vigorously pursued for enabling and enhancing myriad technological developments, including electromagnetic confinement in optically driven cavities^11^,^12^ and, especially, thermal-energy management and conversion^13^,^14^,^15^,^16^,^17^,^18^. Achieving this requires a deep understanding of the profound influence that phonons have on emergent properties in both engineering and advanced materials through the fundamental structure–function relationships at work therein. As such, the formulation of a comprehensive microscopic description of the real-time interaction of propagating modes with individual lattice discontinuities would constitute a significant advance toward ultraprecise directed-energy nucleation and mode guiding in defect-laden materials.

Of particular interest is the development of a detailed understanding of phonon dynamics in nanostructured and disordered semiconducting materials (for example, SiGe alloys and composites)^19^,^20^,^21^, especially those having tunable band gaps, such as the layered transition metal dichalcogenides (for example, WSe_2_, MoS_2_, NbSe_2_ and so on)^5^,^6^,^9^,^10^,^22^. These compounds have generated an enormous amount of interest owing to dramatic differences in, and tunability of, transport properties (thermal and electronic) along different crystallographic directions and with varying numbers of layers. For example, it was shown with WSe_2_ that the cross-plane thermal conductivity could be made several times smaller than the predicted minimum value (indeed, the smallest measured for any fully dense solid) via disordering of the two-dimensional sheets^23^. This was attributed mainly to phonon localization stemming from the incommensurate nature of the individual crystalline WSe_2_ planes, thus restricting these modes from significantly contributing to thermal transport.

To elucidate the dynamics of collective excitations in the presence of individual atomic-scale defects, one must contend with the difficult-to-access spatiotemporal regimes in which these phenomena operate. At the unit-cell level, speed-of-sound phonon wavefronts typically transit such spatial dimensions in less than 100 femtoseconds. The propagating energy causes local bond modulation and a transient elastic deformation of the lattice, briefly re-orienting the atoms in a manner dictated by the symmetry of the mode. Structural dynamics of this nature are amenable to study with ultrafast methods that make use of the dependence of scattering wavevectors on lattice orientation and symmetry^24^,^25^,^26^,^27^,^28^; movement or spacing and symmetry changes of the reciprocal lattice on a fixed Ewald sphere produces a commensurate modulation or re-configuration of the resulting coherent-scattering pattern. In reciprocal space, the dearth of phase information, combined with the probing of lattice motion within material volumes that are large relative to nanoscale discontinuities (for parallel-beam conditions), produces an ensemble-averaged picture of the dynamics, despite being sensitive to symmetry changes^29^,^30^,^31^, bond dilation and breaking^2^,^3^ and atomic-vibration amplitude^32^,^33^,^34^. Conversely, phase information is retained with femtosecond electron imaging^35^,^36^,^37^,^38^, thus enabling spatiotemporal localization of discrete phonon-nucleation events and resolution of propagation dynamics at individual atomic-scale defects.

Here we report direct visualization of the nucleation, evolution and decay of individual acoustic phonons in the vicinity of atomic-scale defects and nanoscale variations in lattice orientation via femtosecond real-space imaging with an ultrafast electron microscope. Experimentally, we observe the launch of individual phonon wavefronts following coherent optical excitation of macroscopically ordered but microscopically disordered materials: here, crystalline tungsten diselenide (WSe_2_) and germanium (Ge). The transient low-energy elastic deformations associated with the propagating waves produce a slight re-orientation of the lattice and thus a localized, rapidly varying Bragg condition. This manifests as coherent diffraction-contrast wavetrains in the real-space images, the nucleation and apparent dynamics of which are found to be strongly dictated by even minor lattice imperfections. Further, we observe that the appearance and directionality of the propagating wavefronts, as seen in the femtosecond bright-field images, are strongly influenced by individual step edges, interfaces and crystal bending. By isolating and quantifying the transient contrast modulation, we extract the spatially dependent vibrational-mode shapes and properties—namely, symmetries, frequencies, phase velocities and decay times—over fields-of-view on the order of the phonon wavelengths. These observations open the way to detailed study of the effects of individual atomic-scale defects, spatially varying lattice orientations and associated strain fields on ultrafast energy propagation in less-than-pristine materials.

## Results

### Overview of WSe_2_ and Ge specimens

**Figure 1:**
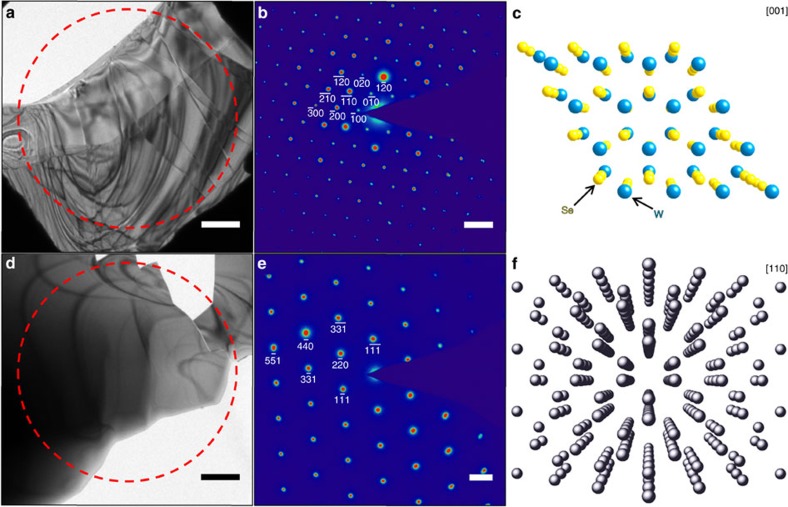
Morphological heterogeneity and atomic-scale order of the WSe_2_ and Ge specimen regions of interest. (**a**,**d**) Bright-field images of (**a**) a WSe_2_ flake and (**d**) a thin Ge crystal. The red, dashed circle denotes the position of the selected-area aperture used to generate the diffraction patterns shown in **b** and **e**. Scale bars, 1 μm. (**b**,**e**) Corresponding selected-area diffraction patterns obtained approximately along the [001] and [110] zone axes for WSe_2_, and Ge, respectively, with several Bragg spots indexed. Scale bars, 5 nm^−1^ (**b**); 2 nm^−1^ (**e**). (**c**,**f**) Crystal structures of (**c**) WSe_2_ and (**f**) Ge, as viewed down the [001] and [110] zone axes, respectively. In **c**: yellow spheres=Se; blue spheres=W, as labelled.

To locate and characterize specific defects and other nanoscale imperfections of interest, the WSe_2_ and Ge specimens were initially surveyed using bright-field imaging and parallel-beam electron diffraction. Static structural and morphological characterization of specific specimen regions of interest, on which subsequent femtosecond electron imaging studies were conducted, are summarized in Fig. 1. As can be seen from the diffraction patterns, the WSe_2_ and Ge specimens are oriented such that the electron beam travels approximately down the [001] and [110] zone axes, respectively (Fig. 1c,f)^39^. Combined, the bright-field images and corresponding diffraction patterns illustrate the macroscopically crystalline but microscopically disordered nature of the regions. From the images, several features of interest for the present study can be identified, which, conversely, are not readily apparent from the diffraction patterns alone. These include step edges and terraces, wrinkles, folds, vacuum-crystal interfaces and cracks. In addition, bend contours are quite prominent and widespread, as are moiré fringes in the WSe_2_ specimen. See the ‘Methods' section below for descriptions of how the specimens were prepared.

### Imaging acoustic-phonon wavefronts and wavetrains

**Figure 2:**
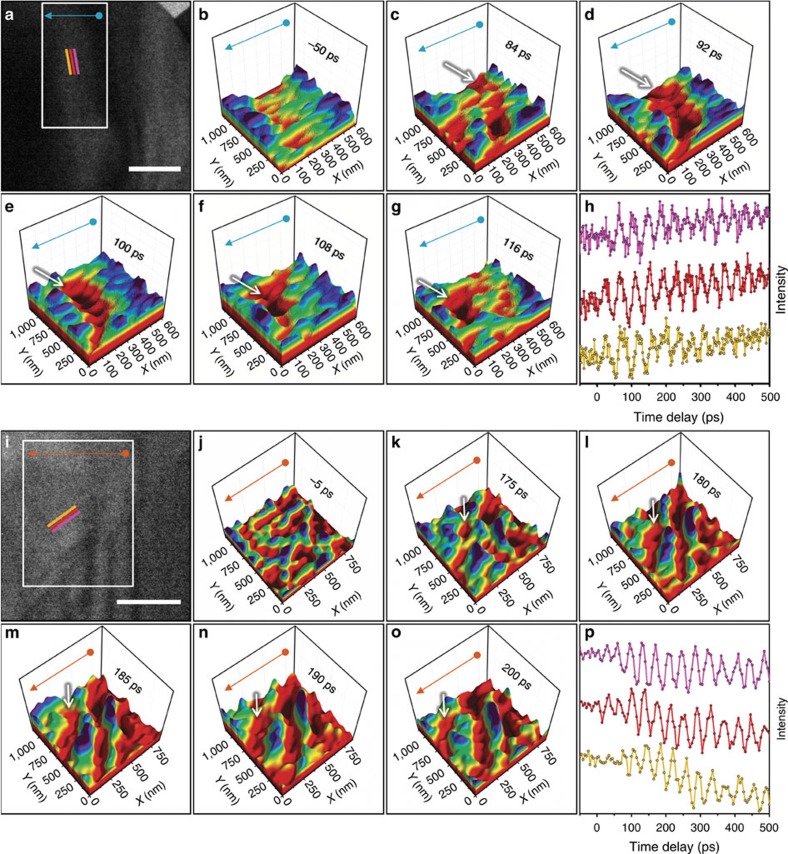
Real-space femtosecond electron imaging of single-phonon wavefronts in Ge and WSe_2_. (**a**,**i**) Bright-field images of the Ge and WSe_2_ regions shown in Fig. 1 and obtained at −50 and −5-ps time delays, respectively. For the Ge experiments, the images were acquired with a 25-kHz repetition rate and a 13-s integration time per frame. For WSe_2_, the images were also acquired with a 25-kHz repetition rate but with an 18-s integration time per frame (see also the captions for Supplementary Videos 1 and 2 for further experimental details). The three coloured lines mark regions from which the mean intensity was quantified and used to generate the time traces in **h** and **p** (described below). The propagation direction is perpendicular to the coloured lines. Scale bars, 500 nm. (**b**–**g**) and (**j**–**o**) Surface plots generated from an image series (region of interest=white rectangles in **a** and **i**) highlighting approximately one period of wavetrain propagation, with a pre-time-zero frame included for reference. Motion of individual wavefronts, which appear as a continuous, deep-red depression, is indicated by the white arrows. The blue and orange arrows map the orientation to the two-dimensional images shown in **a** and **i**. (**h**,**p**) Image-intensity measurements, obtained at the coloured lines in **a** and **i**, as a function of time delay (offset for clarity).

With the applied experimental conditions (stroboscopic femtosecond electron microscopy; see ‘Methods' section), propagating periodic-contrast modulation arising from femtosecond optical excitation was observed in both the specimens (Fig. 2). Owing to the challenge of conveying the behaviour of the observed acoustic-phonon dynamics within a series of static images, the reader is strongly encouraged to view Supplementary Videos 1 through 6, within which the dynamics are striking and more readily apparent. In addition, Supplementary Fig. 1 comprises the same images as shown in Fig. 2, but the display method is different in order to provide additional perspective. From the sequence of femtosecond electron images, phase velocities of 6.5 and 5.5 nm ps^−1^, commensurate periodicities of 40 and 44 ps, and GHz frequencies (Supplementary Fig. 2) were determined for the travelling contrast modulations in Ge and WSe_2_, respectively. This was accomplished by analysing oscillations in the image intensity as a function of both space and time (Fig. 2h,p; see ‘Methods' section). The close correspondence of the phase velocities to the bulk, in-plane speed of sound indicates the contrast dynamics arise from propagating acoustic phonons—major energy carriers emerging from electron–phonon coupling and reflecting the onset of coherent thermal transport. Note that GHz lattice-oscillation frequencies have also been observed in crystalline Si with an ultrafast electron microscope via femtosecond convergent-beam diffraction^40^.

From the image series, it is immediately apparent that the phonon wavefronts do not emerge in a spatially uniform manner. Rather, within the WSe_2_ flake, wavetrains are nucleated at a localized region along a distinct step-edge, and the subsequent propagation direction is perpendicular to the interface (see Supplementary Video 1). The wavetrain emerges in 1 ps (see below), is launched and propagates at a velocity of 5.5 nm ps^−1^ away from the step edge. In the Ge specimen, the appearance and directionality of the propagating phonon wavefronts are seen to follow existing bend contours present in otherwise pristine crystalline regions (see Supplementary Video 2). Also apparent in the video are relatively weak contrast oscillations in the upper-right portion of the frame. These oscillations occur within a relatively pristine region of the Ge specimen (that is, free of significant defects and crystal bending, as indicated by the relatively weak contrast).

### Emergence of a phonon from a step edge

**Figure 3:**
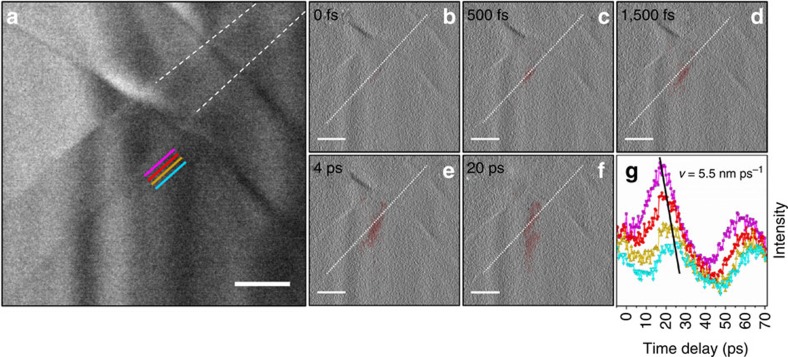
Femtosecond-resolved phonon nucleation and launch at a crystal step-edge. (**a**) Bright-field image highlighting two step-edges (indicated by the partial dashed white lines) in the WSe_2_ flake. The coloured lines represent regions from which the mean intensity was quantified and used to generate the time traces in **g**. The images were acquired with a 50-kHz repetition rate and a 20-s integration time per frame (see also the captions for Supplementary Videos 3 and 4 for further experimental details). (**b**–**f**) Select processed micrographs revealing the nucleation and emergence of a localized phonon wavefront (red). The dotted white lines indicate the position of the step-edge from which the wavefront emerges. (**g**) Intensity measurements obtained at the coloured lines in **a** and plotted as a function of time delay. The slope of the line passing through the peaks of the first oscillation period reflects the wavefront velocity of 5.5 nm ps^−1^. Scale bars, 200 nm.

To precisely resolve the phonon dynamics at an individual step edge, image series of WSe_2_ were acquired with increased magnification and finer temporal sampling (500-fs steps). Within the region of interest highlighted in Fig. 3, the intensity was observed to initially increase at a step edge in the first few-hundred femtoseconds and continue to grow for approximately 10 ps before relaxation via emission of a travelling wave approximately perpendicular to the interface (see Supplementary Videos 3 and 4). The processed difference images (Fig. 3b–f; see ‘Methods' section) and corresponding time-dependent intensity traces (Fig. 3g) display the emergence mechanism of the in-plane acoustic phonons shown in Fig. 2j–o. Notably, the frequency of dynamic intensity at the step edge is in accord with the interlayer echoing of back-and-forth acoustic phonons and resulting oscillating moiré fringes^41^,^42^,^43^ (see also Supplementary Fig. 3). It is therefore likely that the differential stress imparted on the interface by dephasing of the longitudinal *c* axis waves across regions of differing height gives rise to the formation of the in-plane travelling phonons. Systematic studies to probe this and other aspects associated with the dynamic contrast mechanisms are currently in progress and will be described elsewhere.

### Spatially dependent acoustic phonon properties

**Figure 4:**
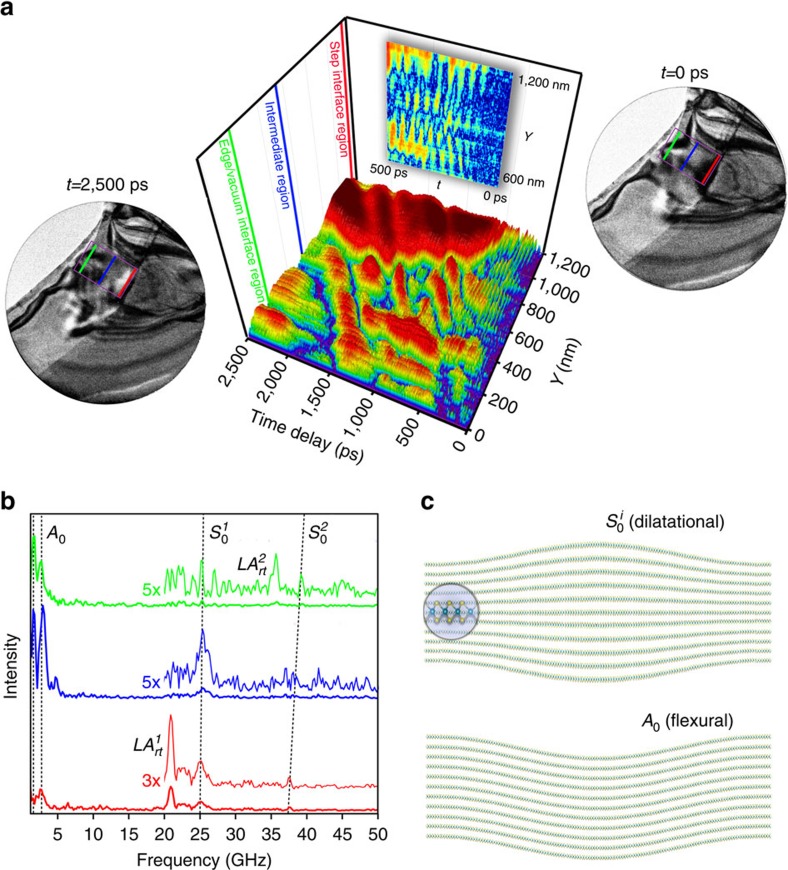
Analysis of distinct localized phonon modes in WSe_2_. (**a**) Surface plot revealing the evolution of two primary modes, as observed in the region of interest highlighted in the accompanying images (*t*=0 and 2,500 ps; purple boxes). The images were acquired with a 25-kHz repetition rate and an 18-s integration time per frame (see also the captions for Supplementary Video 5 for further experimental details). The coloured lines demarcate the specific subregions analysed. The thin, near-vertical streaks predominantly in the sub-500-ps range and spanning the entire step interface region (600 to 1,200 nm; magnified in the inset) are produced by a relatively high-frequency mode, whereas the large amplitude, broad, diagonal features continuing out to the time-window limit are due to a more slowly propagating low-frequency mode. (**b**) Time-domain Fourier transform of traces obtained from the subregions marked by the coloured lines on the surface plot in **a**. The peaks arising from echoing of longitudinal acoustic phonons (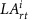) are labelled, while the travelling-wave modes (*A*_0_, 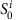) are indicated by the dashed lines, highlighting propagation dispersion (see also Supplementary Fig. 4, which illustrates dispersion observed in the Ge crystal, and Supplementary Video 6, which shows the effects of edge shape on the appearance of propagating diffraction contrast). The superscript *i* indicates waves emanating from the thicker step-edge region (*i*=1) and from that bounded by the crystal-vacuum interface (*i*=2). The spectra are offset for clarity. (**c**) Schematic of the symmetry of the propagating phonon modes with magnified view of a single layer. The dilatational modes (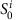, top) occur near 25 and 40 GHz, while the flexural modes (*A*_0_, bottom) range from 2 to 5 GHz.

The information contained in the image series can be further illustrated via a space–time surface plot (see ‘Methods' section). In Fig. 4a, an analysis of contrast dynamics observed in the region between the WSe_2_ crystal–vacuum interface and the step edge is summarized (see also Supplementary Video 5). Each streak corresponds to one period of an acoustic phonon, with the slope and width indicative of phase velocity and frequency, respectively. Such an analysis reveals the presence of multiple modes in this region, with a relatively high-frequency oscillation (phase velocity=5.5 nm ps^−1^) generated during the initial moments of excitation, and slower (0.9 nm ps^−1^), lower-frequency dynamics dominating after a few-hundred picoseconds. Similar spectral features identified with Brillouin light scattering from thin silicon membranes have been attributed to Lamb-wave modes^44^. Analogously here, the confinement of longitudinal acoustic phonons within the specimen thickness gives rise to in-plane propagating modes. These consist of travelling symmetric (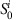) and antisymmetric (*A*_0_) interlayer displacements (Fig. 4c). It has been predicted that such dilatational and flexural acoustic modes significantly influence thermal transport in layered materials^45^.

Inspection of the spatial dependence of the two phonon modes illustrated in Fig. 4 via application of a discrete time-domain Fourier transform in three different spatial regions reveals that both are split into multiple branches, with the degree of excitation dependent on local morphology. A frequency trace near the crystal–vacuum interface returns dominant modes at 36 and 39 GHz (Fig. 4b, green), whereas 21 and 25 GHz are found at the step edge (Fig. 4b, red). In each case, the two dominant peaks correspond to the frequency of the back-and-forth *c* axis acoustic phonons (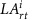) and that of the resulting dilatational Lamb-wave mode (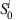). Consequently, the morphologically dependent dispersion likely results from the difference in thickness between the regions from which phonons originate. In addition, the low-frequency flexural mode (*A*_0_) is also split into multiple branches despite emanating from a region of uniform thickness. Although this may occur from phonon interactions with bends and ripples in the flake, analysis of frequency traces obtained from the intermediate section (Fig. 4b, blue) show that the *A*_0_ mode is strongly damped upon reaching the step edge. Such dynamics are the basis of the large disparities in thermal conductivity of layered materials^23^,^45^.

## Discussion

For all the studies reported here, the laser-excitation wavelength used was 515 nm (2.41 eV). This photon energy is significantly larger than the band gaps of both WSe_2_ and Ge (1.6 and 0.66 eV, respectively)^46^,^47^. Accordingly, the roles played by charge-carrier excitation and recombination on localized acoustic-phonon nucleation and launch may be non-negligible. Indeed, the question of why acoustic phonons first appear at specific locations in the material is nontrivial to answer considering the sequence of events that occurs following femtosecond excitation—especially in a disordered material—and the mechanisms of contrast formation in the bright-field images (discussed below). Although this topic is the subject of current systematic studies in our lab, it nevertheless is perhaps worthwhile to draw comparisons to multi-photon pump-probe photoemission electron microscopy. In this variant of photoemission electron microscopy, resonant excitation is used to induce surface-plasmon oscillations and image their launch, propagation and evolution with femtosecond resolution^48^,^49^. An analogous methodology dubbed photon-induced near-field electron microscopy^50^,^51^, which is not reliant on resonant excitation^52^,^53^,^54^, has recently been used to image wave-particle properties of surface plasmon polaritons and induce Rabi oscillations in swift, unbound electron packets^55^,^56^. With the resonant-excitation approach in mind, one can envision femtosecond electron imaging experiments on acoustic-phonon dynamics, where the pump-photon energy is varied with respect to the band gap of the material under study. In this way, one may be able to determine the roles played by the various dynamic and transport phenomena at work.

Beyond the acoustic-phonon excitation and nucleation mechanisms, it will be important to quantify the precise manner in which contrast is formed in the femtosecond electron images. It is well known that deviations of a few milliradians in the local Bragg condition can produce significantly different contrast patterns in bright-field micrographs. Indeed, this sensitivity to local morphology is what enables observation of dynamic contrast from small angular perturbations caused by in-plane propagating waves. It is expected that the nature of the contrast resulting from the acoustic waves is highly dependent on both vicinity to a zone axis and the specific zone axis itself. It is important to note that each of the videos of the WSe_2_ flake presented in this study are acquired at slightly different orientations (due to the variability of sample insertion from one experiment to the next) and in fact show noticeable differences in static diffraction contrast (compare Supplementary Videos 1 and 5). In each case, however, the waves appear to emanate from the same features, and the frequencies and phase velocities extracted are the same. Nevertheless, the exact dependence of specimen orientation with respect to specific dynamic modulations indeed is an open question (and will be the subject of a forthcoming publication).

In conclusion, we have reported the direct, real-space imaging of acoustic-phonon dynamics in macroscopically ordered but microscopically disordered crystalline WSe_2_ and Ge. Via femtosecond electron imaging, we have discovered that phonon nucleation and launch occurs at discrete spatial locations along individual step edges, and that the appearance of coherent, propagating wavefronts are extremely sensitive to the shapes of local strain fields (manifested in bend contours) and vacuum–crystal interfaces. Further, the analysis of picosecond contrast modulation reveals the phase velocities, frequencies and symmetries of the modes, with the spatial and layer-thickness dependence of the oscillations being resolved. We expect that these observations, and the method used, will open the way to the ultraprecise manipulation and control of coherent energy propagation at the atomic scale, with the possibility of exploring the spatiotemporal limits of quantized thermal energy^57^.

## Methods

### Specimen preparation

The WSe_2_ (2-H) flakes were prepared via mechanical exfoliation^58^ of a single-crystal obtained from Nanoscience Instruments. Isolated flakes were transferred to an atomically flat, cleaved (100) NaCl substrate (Ted Pella) before a polymer support film was deposited by drop-casting 20 μl of a solution of 2-wt% polymethyl methacrylate (PMMA) in anisole. The NaCl substrate was etched for 10 to 15 min in a de-ionized water bath, leaving the flakes supported by the PMMA film. The specimens were then positioned on a 2,000-mesh Cu TEM grid via micromechanical manipulation followed by dissolution of the PMMA support film in an acetone bath overnight.

The Ge specimen was prepared via mechanical polishing at a 2° angle from a bulk (110)-oriented, undoped wafer (MTI Corporation) followed by ion milling to electron transparency. The specimen was then epoxy bonded to a Cu slot grid. The irregularly shaped edges of the Ge specimen result from the polishing process as well as non-uniform material removal during ion milling.

### Laser parameters

The Ge and WSe_2_ specimens were optically excited *in situ* with a pump pulse of 270-fs duration full-width at half-maximum and centred at 515 nm (2.41 eV). The pulses were generated with a Yb:KGW (1.03 μm fundamental), diode-pumped solid-state laser and harmonics generation module (Light Conversion; PHAROS and HIRO, respectively). The pulse duration was measured *ex situ* with an in-house-built autocorrelator. The pump fluences incident on the Ge and WSe_2_ were 1.3 and 5.0 mJ cm^−2^, respectively. These fluences were calculated on the basis of a laser spot size of 100-μm full-width at half-maximum, as measured *ex situ* with a beam profiler (Newport LBP-1) using focusing parameters identical to those along the path to the specimen region.

To generate the probing photoelectron packets, the pump line was split, and a portion of the 515-nm pulses was frequency doubled to 257.5 nm and focused into the gun region of the microscope. The experiments highlighted in Figs 2 and 4 of the main text were performed at a repetition rate of 25 kHz, while the higher-resolution scan in Fig. 3 was performed at 50 kHz. In each case, the repetition rates were such that complete mechanical and thermal relaxation was achieved before each subsequent excitation.

### Microscope parameters

All the experiments were performed with a Tecnai Femto ultrafast electron microscope (FEI Company) operated at 200 kV in both thermionic and photoelectron modes. In both modalities, a truncated, 150-μm flat LaB_6_ cathode (Applied Physics Technologies) was used. To capture the greatest number of photoelectrons at the relatively low repetition rates used here^59^, a custom 1,250-μm condenser aperture was used for all the experiments. For all the bright-field experiments, a 40-μm objective aperture was used. For all the selected-area diffraction experiments, a 200-μm projection aperture was used, which collected electrons passing through a 20-μm^2^ area. This same aperture can be seen in the bright-field-imaging Supplementary Videos 1 and 5. The images were recorded with a Gatan Orius SC200B 2,048 × 2,048 CCD camera and with integration times ranging from 13 to 20 s per frame. On the basis of the total electron counts (approximately (1 × 10^8^) to (5 × 10^8^)) acquired with the beam focused to the size of the CCD chip for a given exposure time and repetition rate, it is estimated that 200 to 1,000 electrons per pulse were used for image formation.

### Control experiments

To exclude a host of potential artifacts and undesirable instabilities of the experimental system as the cause of the observed propagating contrast waves, a series of control experiments were performed in duplicate. These were conducted immediately after scans in which the periodic contrast was observed and using the same experimental parameters. (1) A control experiment for specimen drift/tilt was twice performed by acquiring 47 images at a fixed time delay (for example, −100 ps) to replicate the duration of a full-time scan (for example, 20 min). (2) A control experiment for image fluctuations due to potential photoelectron-source instabilities was performed twice by acquiring 50 images over 20 min without specimen excitation. (3) A control experiment for beam instabilities due to movement of the delay stage was performed twice by acquiring 50 images without specimen excitation but still translating the delay stage on the laser table as if a scan (200 ps with 4-ps steps over 20 min) were being performed. (4) A control experiment for the equilibrium thermal effects of pumping the specimen was performed twice by acquiring a series of 50 images over 10 min spanning a delay range of 200 ps but using a thermionic rather than photogenerated electron beam. No propagating contrast waves—indeed, no dynamics of any kind—were observed in the controls.

### Data and image processing

In generating panels (b–g) and (j–o) in Fig. 2 of the main text, several steps were taken in converting the photoelectron images to three-dimensional surface plots. First, difference images were created by taking an average of 10 pre-time-zero images (dubbed the reference image) and subtracting it from each frame in the series. Second, the difference image was modified for contrast and brightness to enhance the features of interest, including passing the images through a 10-pixel Gaussian smoothing filter. Third, each image was levelled by adjusting any pixels with values above 90% or below 10% of the maximum and minimum intensity, respectively, to the 90 and 10% values, respectively. In addition, a one-pixel swath along the edges of each image was set to the 10% intensity value.

The line scans shown in Fig. 2h,p of the main text were generated by first drift correcting the series of images and rotating each frame such that the direction of propagation was horizontal across the field of view. Following this, three adjacent regions measuring 12 × 100 pixels were identified in each image series such that the contrast waves traversed these in succession; the regions were offset in the horizontal direction such that each would sample the contrast wave one after the other. At every time delay, each region was summed in the vertical direction, and the average was determined in the horizontal. Plotting the mean intensities as a function of time delay makes apparent the transitory nature of the waves, as illustrated in Fig. 2 of the main text. Phase velocities were extracted by taking the slope of a line connecting the peaks of the mean intensities and converting the pixel values of each region to a real-space position. The image series from which Fig. 2a–h and Fig. 2i–p of the main text were generated comprise Supplementary Videos 1 and 2.

The panels (b–f) of Fig. 3 in the main text were generated in a similar manner as Fig. 2. A 6-pixel by 6-pixel average filter was applied to difference images created by subtracting an average of 10 pre-time-zero images (that is, the average reference image). The resulting frame was then thresholded at the 80th percentile and placed in the saturation channel of an HSV (hue, saturation and value) image such that the bright, dynamic contrast appears red. To highlight the appearance of contrast at the interface, a 3-pixel by 3-pixel vertical Prewitt-filtered version of the original image was placed in the value channel. The line scans were obtained in the same fashion as for Fig. 2. The image series from which Fig. 3 of the main text was generated comprise Supplementary Video 3, while the processed images comprise Supplementary Video 4.

The space–time surface plot in Fig. 4a of the main text was generated by first rotating each image such that the axis of propagation is oriented directly vertical. A 651-pixel (*Y*) by 100-pixel (*X*) box was selected for the region of interest and median filtered (4-pixel by 4-pixel). Next, the profile for each time delay was created by determining the mean of the 100-horizontal pixels for each of the vertical single-pixel-wide sections and then subtracting the vertical 651-pixel time-zero reference. Thus, the motion appears as diagonal streaks in the surface plot, with the velocity captured in the slope. The frequency traces were created by taking a discrete Fourier transform of a de-trended time trace for various pixels (one at a time). The displayed results represent the average of the Fourier transforms determined over the 20-pixel vertical range.

In generating panels (b–g) and (j–o) in Supplementary Fig. 1, a series of processing steps were performed, with the goal being to produce an additional means for visualizing the propagating contrast waves without the aid of the video format and within which the dynamics are readily apparent. First, the difference images were created by generating an average reference image and subtracting it from each frame in the series. Then, the difference image was modified for contrast and brightness to enhance the features of interest. Next, an HSV image was created by placing the original (at some time delay, *t*) in the hue channel and the difference image (at *t*) in the saturation and value channels. Finally, the colour balance was modified to emphasize the dynamic contrast. Each image within a data set was processed identically.

## Additional information

**How to cite this article**: Cremons, D. R. *et al*. Femtosecond electron imaging of defect-modulated phonon dynamics. *Nat. Commun.* 7:11230 doi: 10.1038/ncomms11230 (2016).

## Supplementary Material

Supplementary Information Supplementary Figures 1-4 and Supplementary References.

Supplementary Movie 1 Propagating phonons in a WSe_2_ flake. Fast-traveling, periodic contrast in the WSe_2_ specimen emanates from both step-edges in the center of the frames and are also seen at the crystal-vacuum interface running from the middle-left to the upper-right. After a few-hundred ps, a slowly-propagating wave can be observed in the center region, which travels from the interface to the step-edge, where it is damped. The images were acquired with a 25-kHz repetition rate and an 18-s integration time per frame. The video illustrates dynamics at 5-ps steps spanning -100 to 1,795 ps (380 total frames) and plays at 40 frames per second (fps) (i.e., dynamics slowed by 5 x 10^9^ times).

Supplementary Movie 2 Propagating phonons in a thin Ge crystal. The transitory nature of the contrast waves is apparent in the lower-central and upper-right portions of the specimen. The edge of the free-standing crystal is located near the bottom of the frame, while the open section in the upper-central region is a punched-out area likely resulting from the mechanical-polishing process. In the lower region of the specimen, the waves appear to change direction soon after time zero; initially, the observed propagation is in a diagonal direction moving to the upper left of the frame before transitioning to right-to-left motion at approximately 140-ps time delay. The images were acquired with a 25-kHz repetition rate and a 13-s integration time per frame. The video illustrates dynamics at 2-ps steps spanning -132 to 468 ps (301 total frames) and plays at 50 fps (i.e., dynamics slowed by 1 x 10^10^ times).

Supplementary Movie 3 Phonon generation and launch at a step-edge in WSe_2_. At higher magnification and finer temporal sampling, as compared to that in Supplementary Video 1, the onset of contrast at a step-edge is observed near the center of the red box. As highlighted in Fig. 3 of the main text, the contrast strength increases along the step-edge over the course of a few ps and then propagates away from the interface until a second cycle occurs 44 ps later. The images were acquired with a 50-kHz repetition rate and a 20-s integration time per frame. The video illustrates dynamics at 500-fs steps spanning -5 to 71 ps (153 total frames) and plays at 50 fps (i.e., dynamics slowed by 4 x 10^10^ times).

Supplementary Movie 4 Processed image series of phonon generation and launch at a step-edge in WSe_2_. By applying the processing steps used in Fig. 3 of the main text to the images comprising Supplementary Video 3, the contrast modulation arising from the generation and launch of a phonon wavefront at a step-edge in WSe_2_ is enhanced. The images were acquired with a 50-kHz repetition rate and a 20-s integration time per frame. The video illustrates dynamics at 500-fs steps spanning -12.5 to 69 ps (164 total frames) and plays at 50 fps (i.e., dynamics slowed by 4 x 10^10^ times).

Supplementary Movie 5 Influence of local specimen morphology on acoustic phonons in WSe_2_. In order to perform a detailed analysis of the observed phonon modes illustrated in Fig. 4 of the main text, a scan spanning 2.5 ns with 2.5-ps time steps (1,000 total frames) was performed. This scan was conducted on the same region as shown in Supplementary Video 1 but shows different contrast patterns due to a slightly-tilted orientation (approximately 2°) from the previous configuration (orientation effects will be the subject of a future manuscript). The relatively fast and slow contrast waves are again observed, and their initial interaction with and behavior due to the specimen morphology are followed in time. In the middle-left of the frame, the moiré oscillations with 44-ps period, discussed in Supplementary Figure 3, are visible. Note that the video shows only a portion of the total 2.5-ns scan in order to keep the file size manageable for publication, and the time stamps are rounded such that the decimal is not observed in the label. The images were acquired with a 25-kHz repetition rate and an 18-s integration time per frame. The video illustrates dynamics at 2.5-ps steps spanning -10 to 1,438 ps (580 total frames) and plays at 40 fps (i.e., dynamics slowed by 1 x 10^10^ times).

Supplementary Movie 6 Influence of local specimen morphology on phonons in Ge. The local morphology of the Ge crystal - most notably the hole positioned near the center of the frame - causes a shaping effect on the acoustic waves following laser excitation. Though not as obvious as in the other videos, alternating contrast bands can be seen encircling the hole and propagating radially outward several hundred nanometers from the crystal-vacuum interface before decaying in approximately 200 ps. The curved appearance of the contrast waves suggests they are generated either at, or are a function of, the crystal boundaries. The images were acquired with a 50-kHz repetition rate and a 15-s integration time per frame. The video illustrates dynamics at 2-ps steps spanning -82 to 366 ps (225 total frames) and plays at 50 fps (i.e., dynamics slowed by 1 x 10^10^ times).
