# Supplementary material for: Femtosecond electron imaging of defect-modulated phonon dynamics
Source: Nat Commun. 2016 Apr 15;7:11230. doi: 10.1038/ncomms11230 (PMC4835536; doi:10.1038/ncomms11230)
Supplement: Supplementary Information — Supplementary Figures 1-4 and Supplementary References. [file ncomms11230-s1.pdf]

## Supplementary Figures

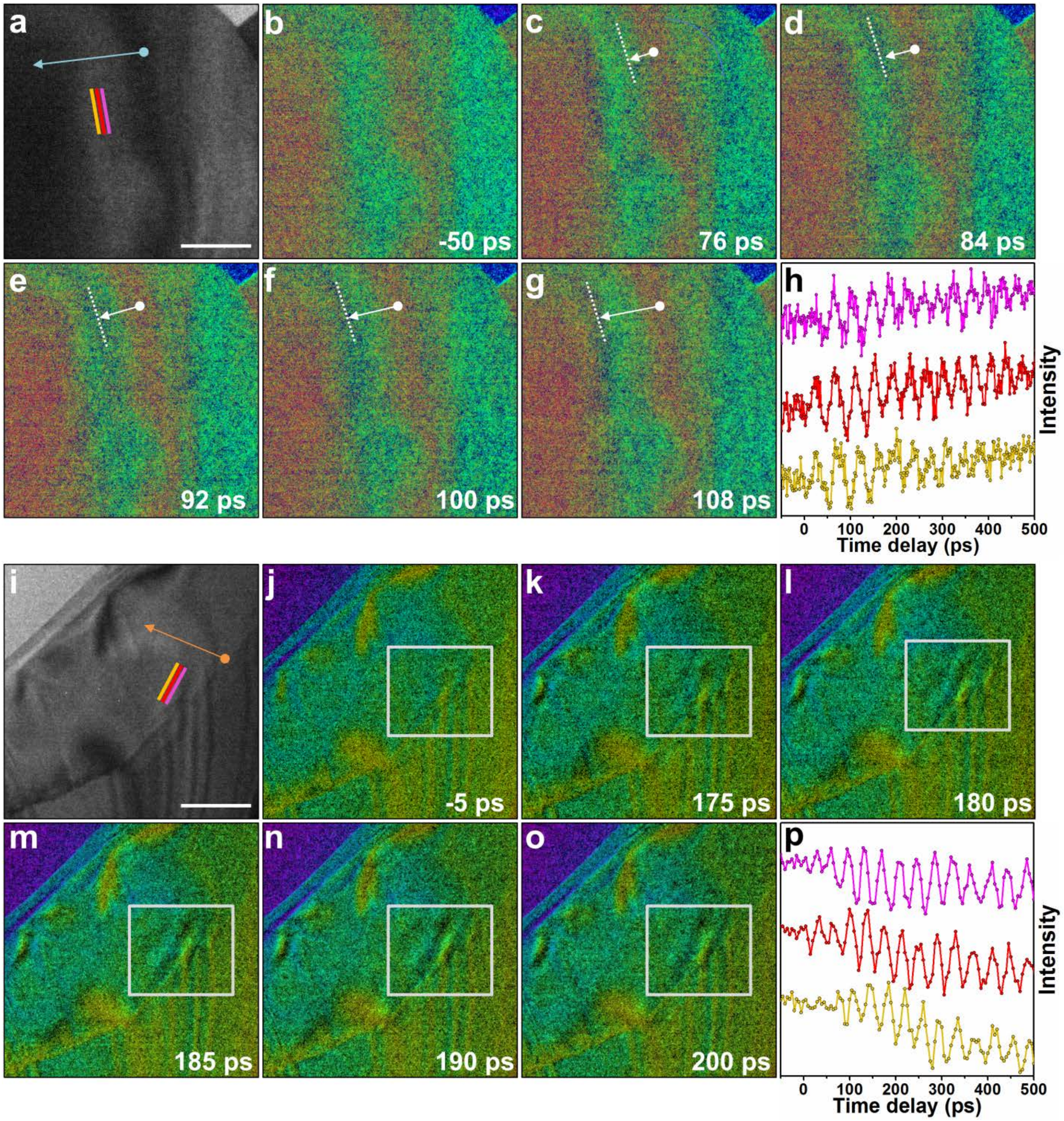


**Supplementary Figure 1. Real-space fs electron imaging of phonon propagation in Ge and $WSe_2$. (a,i)** Bright-field images of the Ge and $WSe_2$ regions shown in Fig. 1 in the main text obtained at -50 and -5-ps time delays, respectively. The three colored lines mark regions from which the mean intensity was quantified and used to generate the time traces in (h) and (p)

(described below). The blue and orange arrows indicate the propagation direction. Scale bars: (a) = 500 nm, (i) = 1 µm. **(b-g) and (j-o)** Select frames from an image series showing approximately one period of phonon propagation. The false coloring was generated by placing a difference frame (*i.e.*, subtraction of a reference image consisting of the average of 10 individual negative-time frames) in the saturation and value channels of an HSV image (see Methods in the main text), while the original micrograph was placed in the hue channel. For Ge, the contrast arising from propagation of the individual phonon appears as a blue band moving from right to left across the light-green region in the center of the positive time-delay images. The peak of the contrast wave is also indicated by the white dotted lines, the movement of which can be observed from the lengthening of the white arrows, which are anchored to a fixed contrast feature in the images. For $WSe_2$, the phonon wavefronts appear as dark and light diagonal stripes within the white box in the false-colored images. All frames show the same particular specimen region of interest. **(h,p)** Image-intensity measurements obtained at the corresponding colored lines in (a) and (i), plotted as a function of time delay. The data are offset for clarity. Note that these are the same images as shown in Fig. 2 of the main text.

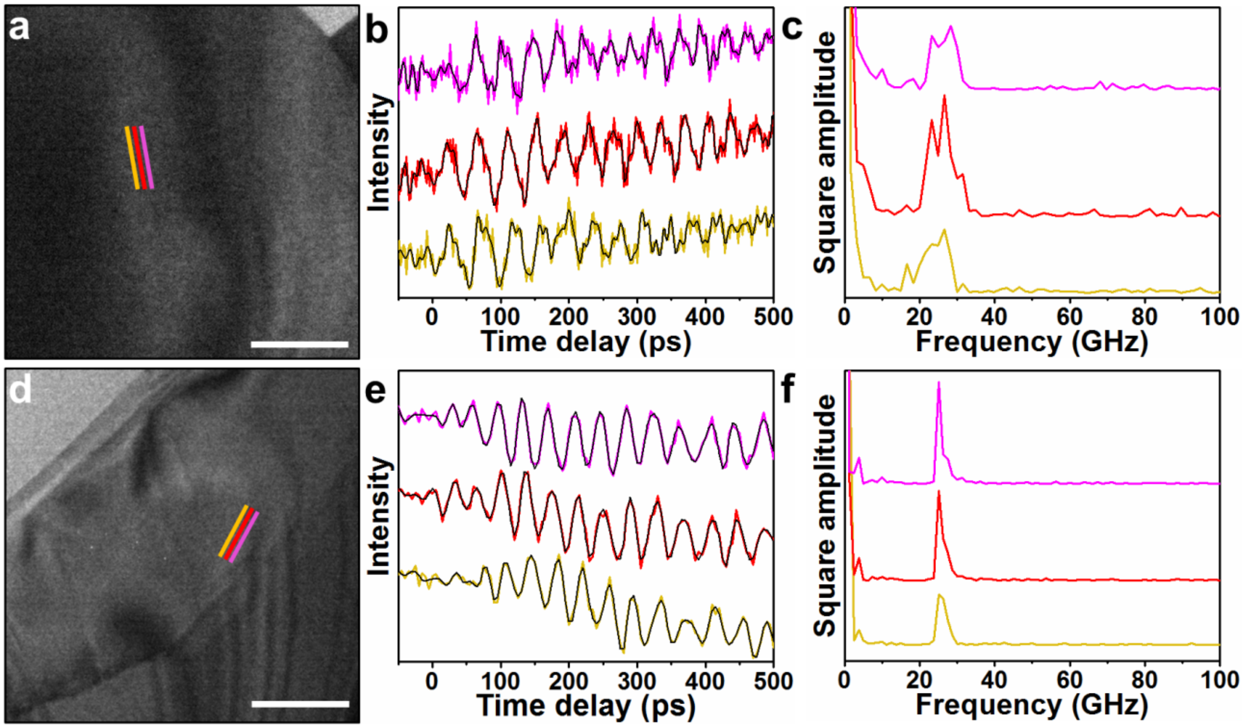


**Supplementary Figure 2. Frequency analysis of phonon propagation in Ge and $WSe_2$.** **(a,d)** Bright-field images of the Ge and $WSe_2$ regions from Fig. 2 in the main text. Scale bars = 500 nm. **(b,e)** Intensity measurements obtained across the colored lines marked in (a) and (d), offset for clarity. Black lines are low-pass filtered data (*i.e.*, filtered above 100 and 50 GHz for Ge and $WSe_2$, respectively). **(c,f)** Time-domain Fourier transform of the time traces in (b) and (e).

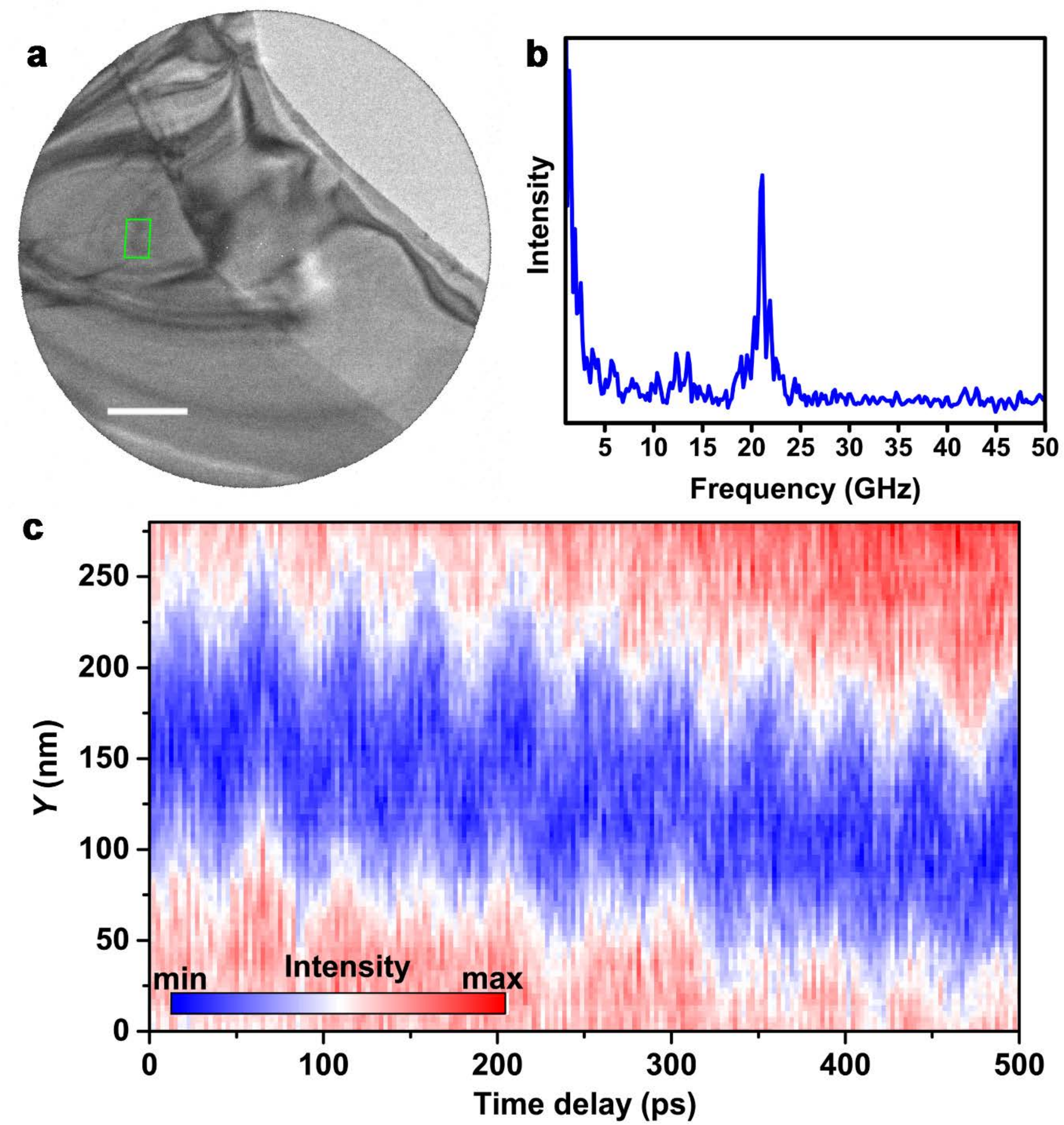


**Supplementary Figure 3. Moiré-fringe dynamics. (a)** Bright-field image of a $WSe_2$ region showing moiré fringes. The green box indicates the region of interest analyzed to generate panels (b) and (c). Note that this is the same specimen region shown in Fig. 4 of the main text. The *xy*-orientation is different because the specimen was removed from the holder following the experiments performed to generate Fig. 4 of the main text and then replaced for the experiments performed to generate this figure. The change in orientation also produced a slight difference in

specimen angle with respect to the incoming photoelectron wavevector. This results in bright-field images (and thus, fs electron images and videos) showing different contrast patterns due to changes in the Bragg condition across the field of view. Detailed studies to quantify this behavior with respect to acoustic-phonon dynamics and fs electron-imaging experiments are currently underway in our lab and will be reported elsewhere. Scale bar = 1 μm. **(b)** Average frequency spectrum generated from 150 pixels perpendicular to the direction of motion within the region of interest. **(c)** Space-time contour plot of the region of interest in (a). Oscillations of the particular moiré fringe (blue) about a fixed spatial position are indicative of the echoing of the *c*-axis phonons against the boundaries of the layered crystal, with the period given by $\tau = 2d v^{-1}$, where $d$ is the crystal thickness and $v$ is the *c*-axis speed of sound[1]. Taking the *c*-axis speed of sound to be $v$ = 1.65 nm ps$^{-1}$ (as measured with picosecond acoustic and interferometry methods)[2] and the thickness to be $d$ = 36 nm yields a period of $\tau$ = 44 ps ($f$ = 22.7 GHz).

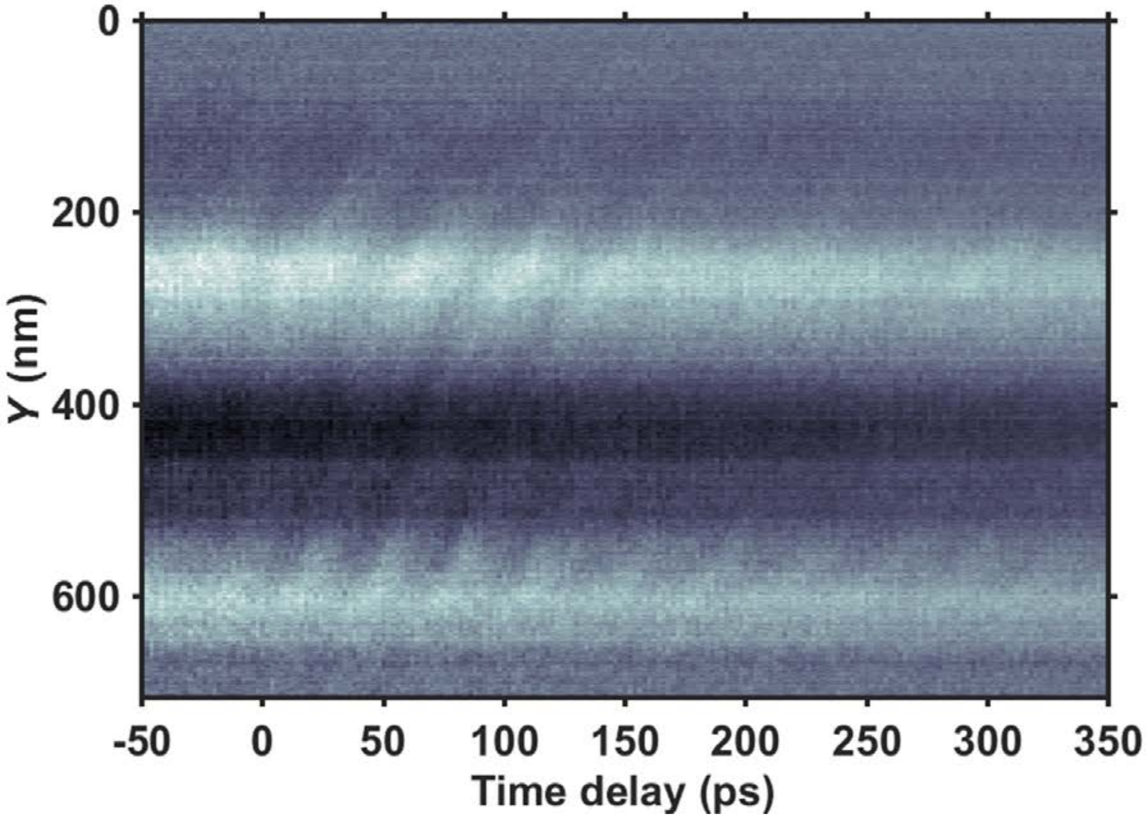


**Supplementary Figure 4. Contour plot of Ge phonon propagation, dispersion, and decay.** Shown are the spatially-dependent contrast waves as a function of time delay. The images from which this dataset originates are the same as those used to create Fig. 2(a-h) in the main text and comprising Supplementary Video 2. The frequency of the bands shown here is centered at 25 GHz, as determined from the discrete Fourier transform in Supplementary Figure 2.

## Supplementary References

1. Park H. S., Baskin J. S., Barwick B., Kwon O.-H. & Zewail A. H. 4D ultrafast electron microscopy: Imaging of atomic motions, acoustic resonances, and moiré fringe dynamics. *Ultramicroscopy* **110**, 7-19 (2009).

2. Chiritescu C., Cahill D. J., Nguyen N., Johnson D., Bodapati A., Keblinski P. & Zschack P. Ultralow thermal conductivity in disordered, layered $WSe_2$ crystals. *Science* **315**, 351-353 (2007).
